# Influence of water quality on diversity and composition of fungal communities in a tropical river

**DOI:** 10.1038/s41598-018-33162-y

**Published:** 2018-10-04

**Authors:** Mabel Patricia Ortiz-Vera, Luiz Ricardo Olchanheski, Eliane Gonçalves da Silva, Felipe Rezende de Lima, Lina Rocío del Pilar Rada Martinez, Maria Inês Zanoli Sato, Rodolfo Jaffé, Ronnie Alves, Simone Ichiwaki, Gabriel Padilla, Welington Luiz Araújo

**Affiliations:** 1 NAP-BIOP, LABMEM, Department of Microbiology, Institute of Biomedical Sciences, University of São Paulo, Av. Lineu Prestes, 1374, Ed. Biomédicas II, Cidade Universitária, São Paulo, SP Brazil; 2 Instituto Tecnológico Vale – Desenvolvimento Sustentável. Rua Boaventura da Silva, 955, Nazaré, 66055-090 Belém, PA Brazil; 3 Department of Environmental Analysis, Environmental Company of São Paulo State (CETESB), Av. Prof. Frederico Hermann Jr., 345, São Paulo, SP Brazil

## Abstract

Freshwater fungi are key decomposers of organic material and play important roles in nutrient cycling, bio-remediation and ecosystem functioning. Although aquatic fungal communities respond to pollution, few studies have quantitatively assessed the effect of freshwater contamination on fungal diversity and composition; and knowledge is scarcer for tropical systems. Here we help fill this knowledge gap by studying a heavily-contaminated South American river spanning a biodiversity hotspot. We collected 30 water samples scattered across a quality gradient over two seasons and analyzed them using Terminal Restriction Fragment Length Polymorphisms (T-RFLP) coupled with 454 Pyrosequencing. Using T-RFLP we identified 451 and 442 Operational Taxonomy Units (OTUs) in the dry and rainy seasons respectively, whereas Pyrosequencing revealed 48,553 OTUs from which 11% were shared between seasons. Although 68% of all identified OTUs and 51% of all identified phyla remained unidentified, dominant fungal phyla included the Ascomycota, Basidiomycota, Chytridiomycota, Glomeromycota, Zygomycota and Neocallimastigomycota, while *Calcarisporiella*, *Didymosphaeria*, *Mycosphaerella* (Ascomycota) and *Rhodotorula* (Basidiomycota) were the most abundant genera. Fungal diversity was affected by pH and dissolved iron, while community composition was influenced by dissolved oxygen, pH, nitrate, biological oxygen demand, total aluminum, total organic carbon, total iron and seasonality. The presence of potentially pathogenic species was associated with high pH. Furthermore, geographic distance was positively associated with community dissimilarity, suggesting that local conditions allowed divergence among fungal communities. Overall, our findings raise potential concerns for human health and the functioning of tropical river ecosystems and they call for improved water sanitation systems.

## Introduction

A major focus of freshwater ecology has been to understand the processes that shape microbial biodiversity in aquatic ecosystems. Microbial communities associated with freshwater environments are composed of a diverse set of bacteria, fungi and archaea, responsible for key processes underpinning ecosystem functioning, such as carbon cycling, biological nitrogen fixation, denitrification, methane production, sulphate reduction, and transformation of metals and various molecules^1,2^.

The disposal of untreated effluents can impact microbial diversity of natural environments, consequently affecting the functioning and health of aquatic environments. Studies have demonstrated that variation in local environments and biotic interactions can shape the structure of microbial communities in fluvial networks^3^. However, microbial diversity and community composition can also be affected by water pollutants, so they have been proposed as sensitive indicators of ecosystem health^4^. The contamination of natural water bodies with industrial, agricultural or urban wastewater can potentially modify composition, structure and microbial activity on a local and global scale, affecting aquatic life and soil fertility^5,6^. Drastic changes in the structure and composition of these communities may thus result in unexpected changes in nutrient flux^7^. Changes in environmental conditions can also cause the extinction of certain taxonomic groups with key ecological functions within the microbial consortia^8^, as well as the spread of potentially pathogenic microbes^9,10^.

Water contamination is also known to influence diversity and composition patterns in aquatic fungal communities^4,11^. For example, fungal communities are profoundly influenced by the amounts of nutrients dissolved in the water^7,12,13^. Nevertheless, most environmental microbiology sequencing studies have focused on prokaryotic organisms, and microbial eukaryotic communities have been largely neglected^14^.

Fungi are among the least-studied groups of aquatic microorganisms, because of difficulties inherent in their culturing and DNA sequencing. Nevertheless, aquatic fungal communities are known to contribute to organic matter mineralization and cycling. Aquatic fungi produce hydrolytic enzymes that degrade many compounds^11,15,16^, thus contributing to the purification of aquatic environments^17,18^. Additionally, they have a high metabolic capacity for carbon capture, and are believed to be key elements in the carbon cycle and regulators of global climate^19^.

In the present study, we help fill the substantial knowledge gap regarding the influence of anthropogenic disturbances on aquatic fungal communities. Our case study involved the fungal community of the Tietê River (Brazil), which is born in preserved area of the Atlantic rain forest, a biodiversity hotspot^20^, and then runs through the city of São Paulo, one of the world’s largest metropolises (>25 million people). While the native vegetation along the Tietê River’s path has gradually been replaced by pastures and diverse crops, industrial and urban areas^21^, untreated effluents have been released into the Tietê River and its tributaries, increasing the level of organic matter and completely changing the river’s characteristics. Nevertheless, a few kilometers downstream the city of São Paulo, the river begins a self-purification process and its water recovers characteristics similar to those observed in the source.

Due to the lack of knowledge regarding the impact of water contaminants in this environment and the huge microbial diversity found in similar tropical ecosystems, many microbial species with potential biotechnological applications are likely to have remained unidentified and may even become extinct before they are described. We thus aimed to answer the following questions: What is the distribution and diversity of fungi along the Tietê River and its tributaries? Are fungal diversity and community composition affected by seasonality and water quality? We expected higher diversity in less contaminated areas, with changes in community structure led by the disappearance of the most susceptible OTUs. Furthermore, we predicted a decrease in community similarity with increasing geographic distance. Finally, we expected higher diversity during the rainy season.

## Material and Methods

Two complementary approaches were performed to analyze the influence of water contamination on aquatic fungal communities of the Tietê River. Samples from 30 locations were collected along the entire river basin, and the Terminal Restriction Fragment Length Polymorphisms (TRFLP) technique was performed to evaluate broad diversity patterns. Seven distant locations, representing a gradient of water quality, were then selected based on these first results, and Roche 454 pyrosequencing was performed to determine taxonomic identity and fine composition patterns.

### Sample collection and DNA extraction

**Figure 1:**
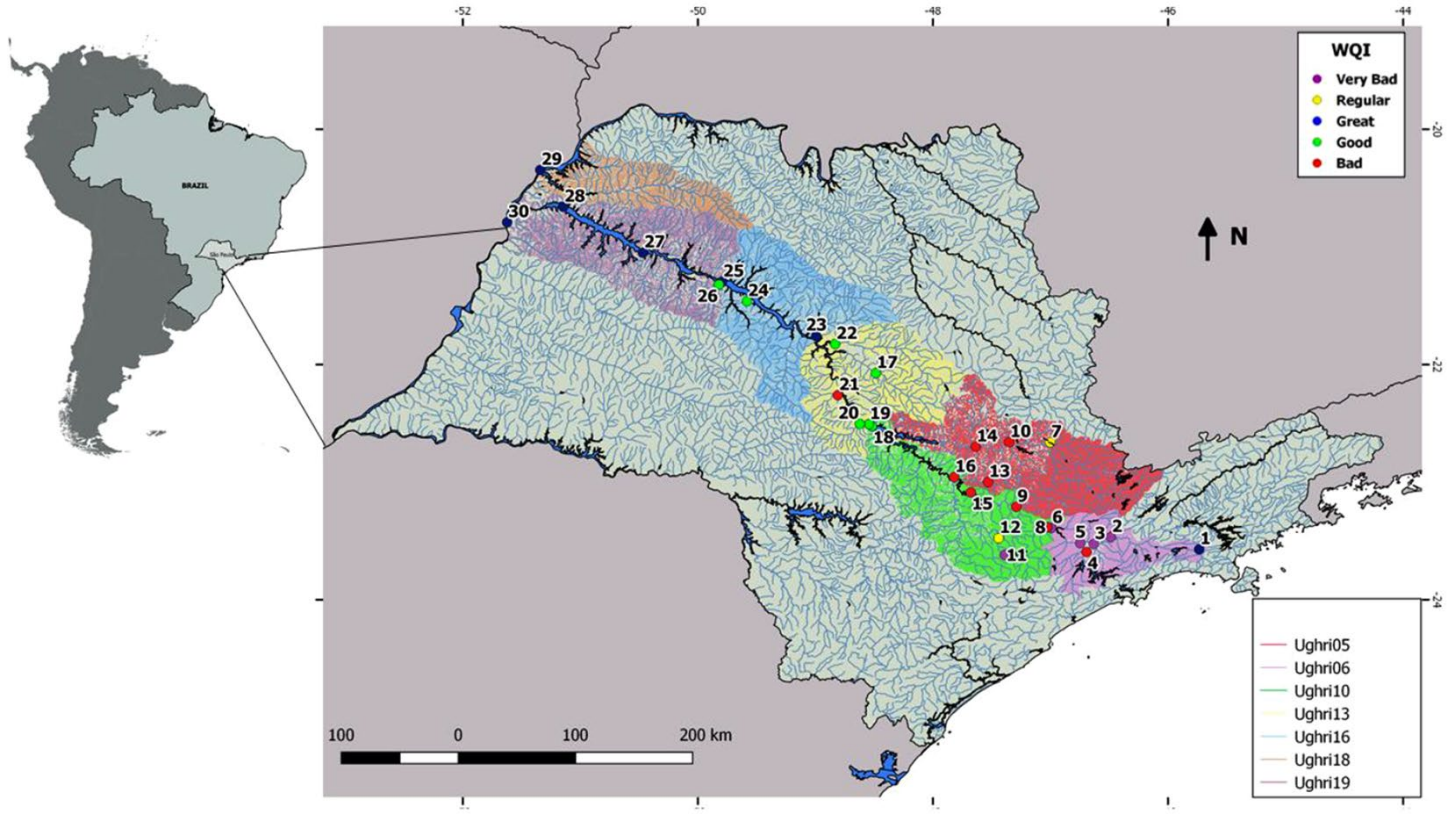
Map of the Tietê River showing all sampling sites. The Water Quality Index (WQI) for each water sample is shown in colors: blue = Great, green = Good, yellow = Regular, red = Bad, violet = Very bad.

Water samples were provided by the São Paulo’s State Environmental Company (CETESB), who also evaluated physicochemical parameters and determined a Water Quality Index (WQI). This WQI integrates biochemical oxygen demand (BOD), total phosphorus (total P), sodium (Na), potassium (K), ammoniacal nitrogen (NH_4_^+^), total iron (total fe), total aluminum (total Al), total organic carbon (TOC), dissolved iron (dissolved Fe), dissolved oxygen (DO), pH, temperature, conductivity, nitrate (NO_3_^−^) and turbidity into a single index that ranges between 0 and 100: Great (79–100), Good (51–79), Regular (36–51), Bad (19–36) and Very bad (0–19). Physicochemical data were retrieved from CETESB reports and are available on the company’s website (www.cetesb.sp.gov.br)^22^. Samples were collected during the dry season in 2013 (average rainfall was 44.72 mm)^22^ and the rainy season in 2014 (average rainfall was 170.42 mm)^22^. A total of 30 water samples were collected in triplicate in both seasons (Fig. 1). Sampling sites were chosen to represent the most contrasting areas according to prior physicochemical characterizations of the Tietê River^22^. Five liters of water from each sampling site were collected and maintained in sterile plastic containers. The samples were then stored at 4 °C until the filtering process that occurred no later than 48 hours after sampling.

Three replicates of one liter of water from each sample were then filtered through 1.2 μm Millipore® GS membranes of cellulose ester (47 mm diameter, white, smooth - ©Merck KGaA, Darmstadt, Germany). Total DNA was extracted from the membranes using the Power Soil DNA Isolation Kit® (MoBio Labs, Inc. Solana Beach, USA) according to the manufacturer’s instructions. To assess extraction success, the internal transcribed spacer (ITS) region of fungal ribosomal RNA was amplified using the primers ITS1 and ITS4, and visualized on a 1.5% agarose gels in TAE buffer (400 mM Tris, 20 mM glacial acetic acid, 1 mM EDTA).

### Terminal Restriction Fragment Length Polymorphism (TRFLP)

The internal transcribed spacer (ITS) ribosomal region was amplified using the primers FAM-ITS1F (5′TCCGTAGGTGAACCTGCGG3′) and ITS4r (5′ TCCTCCGCTTATTGATATC 3′). PCRs were ran employing 25 µl reaction volumes containing: 12.5 µL of Go Taq® G2Hot Start Green Master Mix, 0.1 µL 10 µM of each primer, 2.0 µL of DNA and 10.4 µL nuclease-free water. PCR cycles were programed as follows: 94 °C for 1 minute and 30 seconds followed by 13 cycles of 94 °C for 35 seconds, 55 °C for 55 seconds, 72 °C for 45 seconds, 13 cycles of 94 °C for 35 seconds, 5 °C for 2 minutes, 72 °C for 45 seconds, 9 cycles of 94 °C for 35 seconds, 55 °C for 3 minutes, 72 °C for 45 seconds, followed by a final extension at 72 °C for 10 minutes. DNA and amplicons were visualized on 1.5% agarose gels in TAE buffer.

PCR products were purified and digested with 5 U/µL of restriction enzymes HaeIII at 37 °C for 3 hours. Quality and concentration of these purified PCR products were determined using a NanoDrop Spectrophotometer (Thermo Scientific). The terminal fragments were then analyzed on a Genetic Analyzer 3500xL equipment (Applied Biosystems, Foster City, CA, USA) at EMBRAPA’s Environmental Microbiology Laboratory Environment (Jaguariúna, São Paulo). The program GeneMapper® 4.1 (Applied Biosystems) was used to quantify electropherogram data and generate OTU profiles. Peaks with less than 100 fluorescence units and fragments smaller than 50 bp were excluded from statistical analyses, which were performed using PAST 2.14 (Copyright Hammer and Harper 1999–2012), Canoco for Windows 4.51 (Copyright C 1997–2003 Biometris) and R 2.15.1 (Copyright C 2012 The R Foundation for Statistical Computing). We initially visualized the data using principal component analysis (PCA), aiming to group samples by WQI. We then regressed community with environmental data using redundancy analysis (RDA).

### Pyrosequencing

Based on the T-RFLP diversity estimates and water quality assessments made by CETESB, we selected seven sites scattered across our study area that captured the full range of biological and environmental variation and used them to run deeper analysis using 454 pyrosequencing. We selected three samples within the city of São Paulo that were exposed to domestic pollution (samples 4, 5 and 8). Two additional samples were exposed to industrial effluents (samples 15 and 16) and another two (17 and 26) were located much further downstream, where natural remediation processes have improved quality levels substantially^22^.

We used next-generation amplicon sequencing of the ITS-1 region, the 5.8 S rRNA gene and the ITS-2 region to describe the aquatic fungal community. Samples were ran on a 454 GS FLX platform with titanium chemistry (Roche, Switzerland). This platform was chosen aiming to cover the entire ITS region^23^. We prepared, purified, and quantified PCR reactions in triplicate using the ITS1F and ITS4 primers with multiplex identifier (MID) barcodes. Equimolar mixing of the samples prior to sequencing was performed by DSMA Biotecnologia (Mogi das Cruzes, SP, Brazil).

Bioinformatic analyses were performed using the Quantitative Insights Microbial Ecology (QIIME v. 1.9.1) pipeline. Initially, sequences were de-multiplexed, and primers and barcodes were removed. Sequences smaller than 200 bp and those with a mean quality score below 25 were eliminated. High quality sequences were grouped against the reference database UNITE/QIIME 12_11, using BLAST with a 97% similarity threshold^24^ and OTUs were chosen using an open-reference OTU picking protocol. The representative OTUs were finally selected and taxonomic identification was performed using BLAST.

Fungal diversity was estimated using the Shannon’s diversity index (*H’*). To evaluate changes in community composition, we ran a non-metric multidimensional scaling analysis (NMDS) and used the first two axes as synthetic variables describing community composition. To assess the effects of water quality and seasonality on fungal diversity and composition patterns, we followed a model-selection approach. We ran linear mixed effect models containing diversity or composition as response variables, all physicochemical parameters and collection season as predictor variables and collection site as a random effect. This allowed accounting for data dependencies (triplicates at each sampling site) as well as spatial autocorrelation. We used the *lme4* R package^25^ to build full models for each response variable (diversity, first and second NMDS axes) and compared all models containing non-collinear predictors (*r* < 0.6) using the *dredge* function from the MuMIn (v1.4) package (https://github.com/rojaff/dredge_mc ^26^). Model selection was performed using the Akaike Information Criterion (AIC), and models with ΔAIC ≤ 2 were included in the set of best models. Model averaging across the set of best models was used to compute parameter estimates that accounted for uncertainty in model selection^27^.

Finally, we assessed whether community dissimilarity increased with the geographic distance separating samples. To do so, we employed the *geosphere*^28^ and *vegan*^29^ packages to calculate all pairwise geographic and dissimilarity (Bray-Curtis) distances and perform a Mantel test for each season separately (all R scripts and data files can be found in Supplementary Data S1).

## Results

### Physicochemical water parameters

Samples collected in both seasons were grouped according to the WQI retrieved from the CETESB Report^22^ (Fig. 1). Physicochemical parameters and WQI for all samples are shown in Table S1 and Figure S1. Our PCA analyses revealed how CETESB’s WQI grouped samples with different physicochemical characteristics (while the first PCA axis explained 69.7% of total variance, the second axis explained a further 10.3%; Figure S2). Whereas high levels of DO seemed to be associated with Good and Great WQI samples, higher NH_4_, total P, TOC and temperature were associated with Bad and Very Bad WQI samples (Fig. S2).

### Terminal Restriction Fragment Length Polymorphism (TRFLP)

We identified a total of 451 OTUs in the dry season and 442 OTUs in the rainy season. OTUs present in more than 70% of samples were defined as “core community” and were represented by six and nine OTUs in the dry and rainy seasons, respectively. Only three (0.7%) core OTUs were present in both seasons.

Most fungal OTUs were found in Good or Great WQI samples (Fig. S3). From the 451 OTUs obtained in the dry season, 83 (18.40%) were only found in Good and Great samples, while 46 (10.19%) were exclusive to Bad and Very Bad samples, and 70 (15.52%) OTUs were present in all WQI categories. From the 442 OTUs found in the rainy season, 68 (15.38%) were found in Good and Great samples, 45 (10.18%) were exclusive to Bad and Very Bad samples, and 96 (21.72%) occurred in all WQI categories. These results thus demonstrate that some OTUs are water quality-specific (Fig. S3).

**Figure 2:**
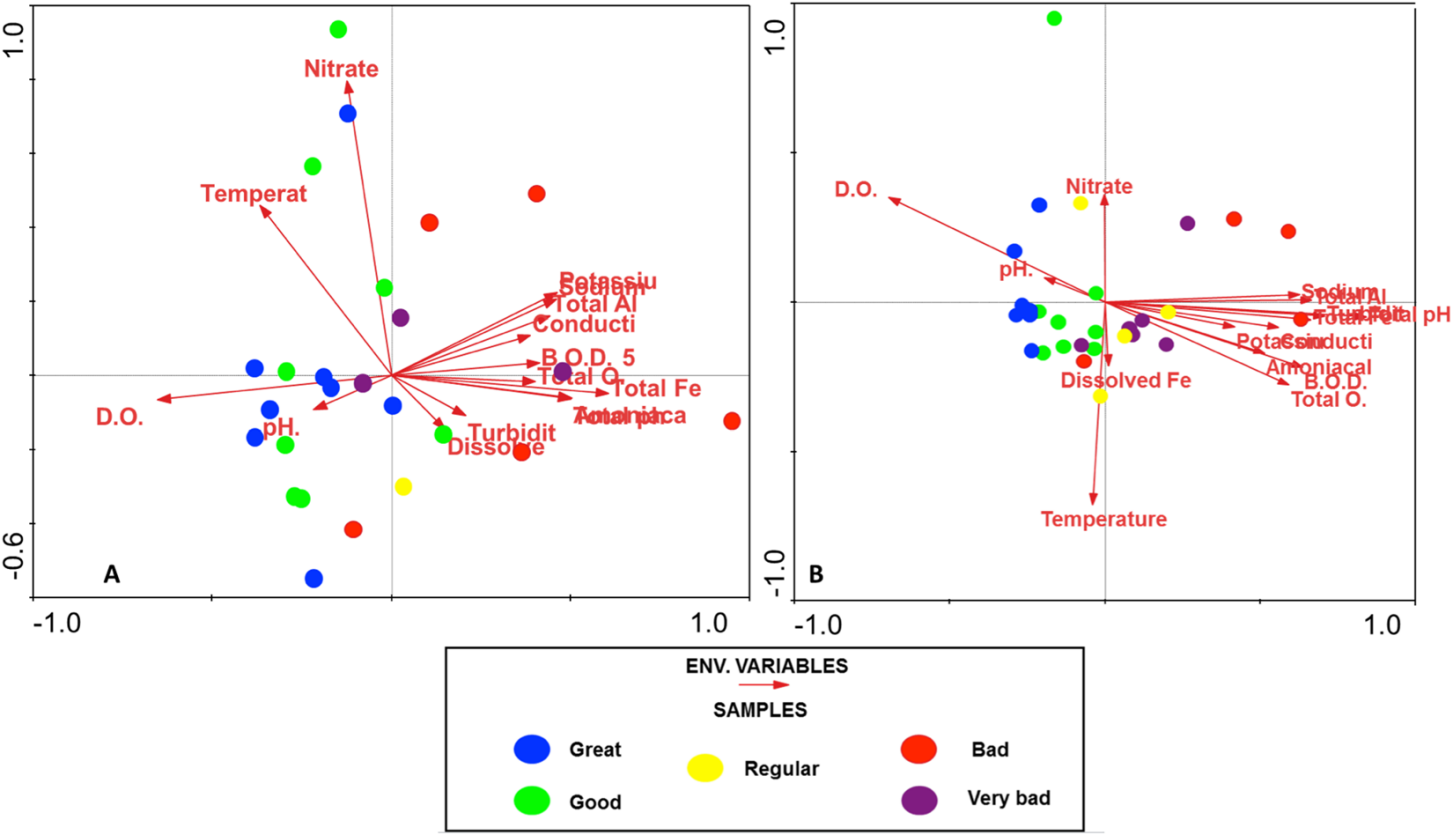
Redundancy analysis (RDA) of the T-RFLP matrix and water quality index. Detrended Correspondence Analysis (DCA) of the fungal communities during the dry (**A**) and rainy (**B**) season. The colored circles indicate the Water Quality Index (WQI): blue = Great, green = Good, yellow = Regular, red = Bad, violet = Very bad.

Redundancy analysis (RDA) revealed that physicochemical parameters grouped samples to some extent (Fig. 2). During the dry season (Fig. 2A) DO, NO_3_^−^, and pH seemed predictors of community structure, whereas pH, temperature, NO_3_^−^ and DO were more important predictors in the rainy season (Fig. 2B). Finally, we observed greater differences in community composition in the dry than in the rainy season.

### Pyrosequencing

**Figure 3:**
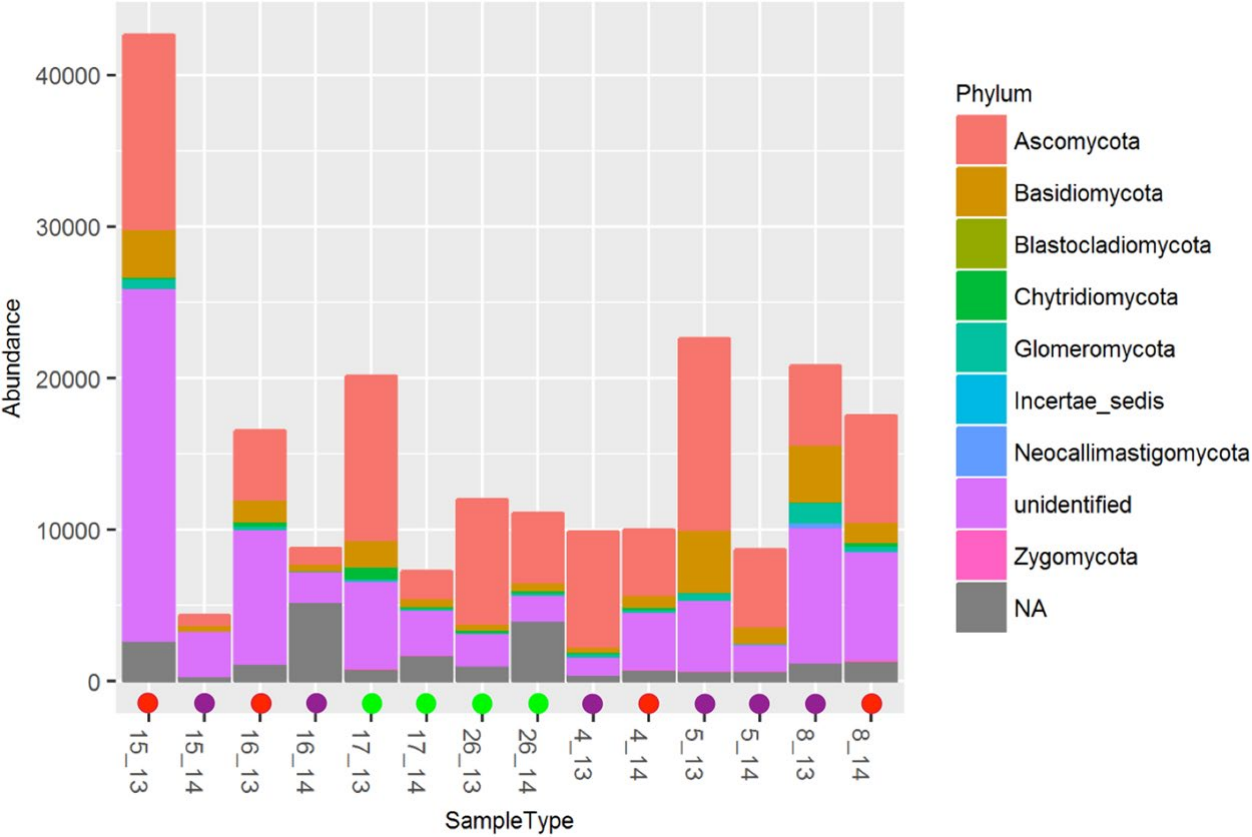
Relative abundance of fungal phyla across sampling locations in the Tietê River. NA: not applicable. The colored circles indicate the Water Quality Index (WQI): blue = Great, green = Good, yellow = Regular, red = Bad, violet = Very bad. The numbers represent sample locations and sampled season (13: dry and 14: rainy season).

Based on QIIME and BLAST, we identified 48,553 OTUs, belonging to several phyla. These included Ascomycota, Basidiomycota, Chytridiomycota, Glomeromycota, Zygomycota and Neocallimastigomycota (Fig. 3). The dominant classes were Sordariomycetes (9,69%), Dothideomycetes (7,59%), Agaricomycetes (6,00%) and Saccharomycetes (4,18%; Figure S4), and the dominant genera included *Calcarisporiella* (Endogonales order), *Didymosphaeria* (Pleosporales order) and *Mycosphaerella* (Capnodiales order) in the Ascomycota phylum and *Rhodotorula* (Sporidiobolales) in the Basidiomycota phylum. Many taxa nevertheless remained unidentified, comprising 68% of all identified OTUs and genera, 65% of the identified families, 60% of identified orders, 60% of identified classes, and 51% of all identified phyla (Figs 3 and S4). Only 11% of all identified OTUs were present in both seasons.

**Figure 4:**
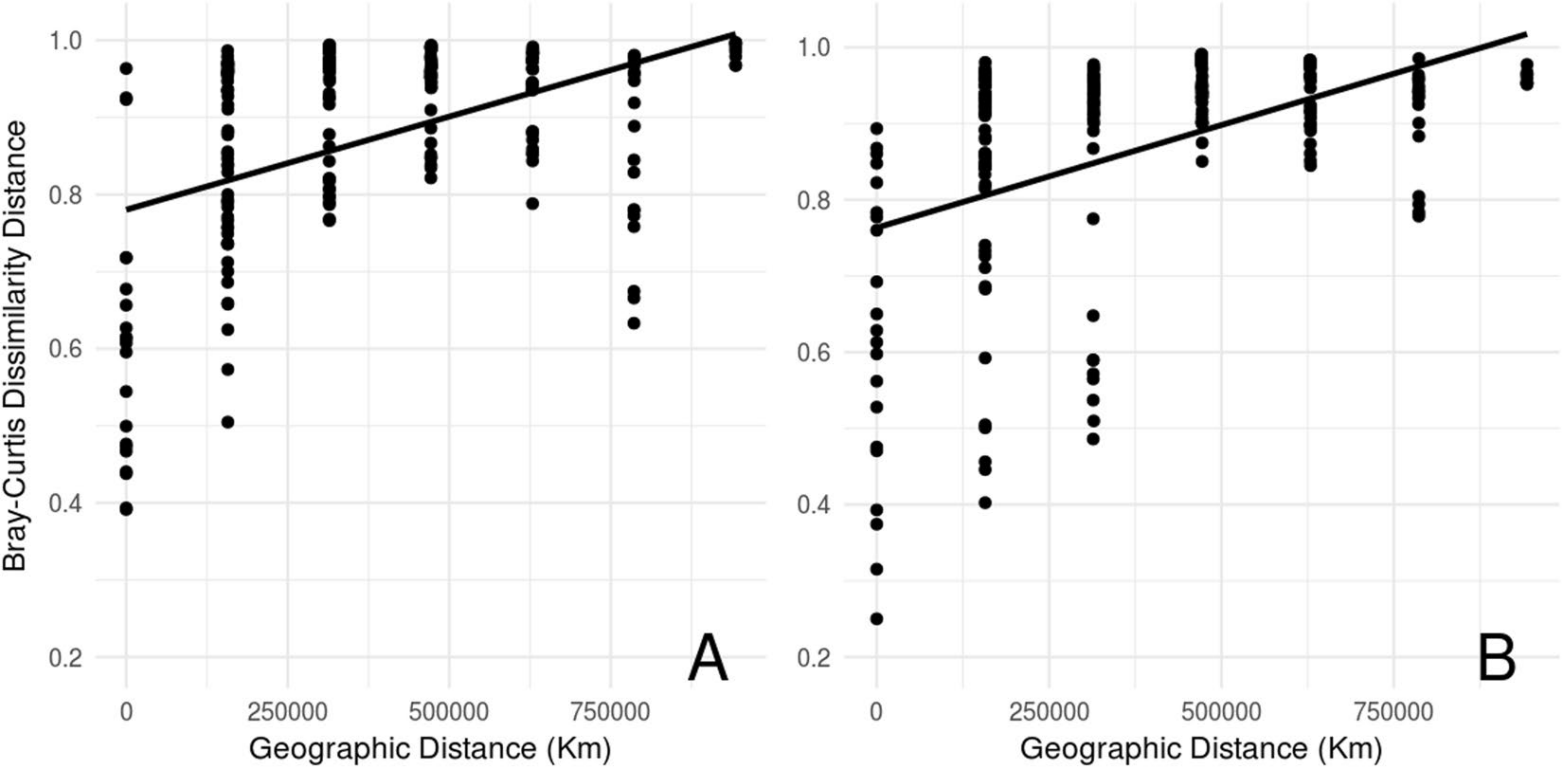
Relationship between fungal community dissimilarity (Bray-Curtis distance) and geographic distance during the dry (**A**) and rainy (**B**) season. Fungal communities showed increasing dissimilarity as the geographic distance separating them increased (Mantel test r = 0.3343, p = 0.001).

We found that pH and dissolved Fe were the environmental parameters that best explained diversity patterns (Table 1). Whereas we found a positive association between fungal diversity and pH, higher iron concentrations were associated with lower diversity. Furthermore, the best models explaining changes in the fungal community composition included BOD, pH, total Al, total Fe, DO, NO_3_^−^, TOC and seasonality (Table 1). When total Al and pH decreased, and BOD and total Fe increased, fungi belonging to Pleosporaceae, Trichocomaceae and other unidentified groups were found to be more abundant, whereas other unidentified groups became dominant in the opposite conditions (Table 1). On the other hand, when NO_3_^−^, total Fe and rains increased and DO and TOC decreased the families Peniophoraceae, Parmeliaceae, Meruliaceae and Nectriaceae were found in higher abundance, while the same was true for unidentified fungi under the opposite conditions (Table 1). Finally, in both seasons, dissimilarity between fungal communities significantly increased with geographic distance (Mantel test r = 0.3343, p = 0.001) (Fig. 4).

**Table 1:** Summary statistics for the best models describing fungal diversity and composition along the Tietê River.

Response	Predictor	Estimate	SE	z/t-value	P
Diversity	Dissolved Fe	−0.3183	0.1542	2065	0.04*
	pH	0.6360	0.1562	4072	<0.001***
	Total P	−0.2935	0.1516	1936	0.05
	Total Al	−0.2961	0.1853	1598	0.11
	Total Organic C	−0.2379	0.1722	1382	0.17
	Seasonality (rain)	0.5274	0.3160	1669	0.09
	Total Fe	0.1331	0.1462	911	0.36
NMDS1	B.O.D	0.23154	0.05465	4237	0.002**
	pH	0.40424	0.04795	8431	<0.001***
	Total Al	−0.27600	0.05629	−4903	<0.001***
	Total Fe	0.33301	0.05057	6585	<0.001***
NMDS2	Amoniacal N	0.31497	0.18613	1692	0.09
	D.O	−0.28334	0.08388	3378	<0.001***
	Nitrate	0.46481	0.10547	4407	<0.001***
	Total Organic C	−0.29615	0.07715	3839	<0.001***
	Seasonality (rain)	0.47151	0.12695	3714	<0.001***
	Conductivity	0.16575	0.11744	1411	0.16
	Total Fe	0.24132	0.06558	3680	<0.001***
	Temperature	0.10733	0.06449	1664	0.10

Model-averaged coefficients based on the suit of best models (ΔAIC ≤ 2) and dry season used as reference level.

## Discussion

In this study, we analyzed water samples collected in the Tietê River along a pollution gradient during two different seasons. T-RFLP and 454 pyrosequencing analysis allowed the identification of fungal community structure and the assessment of community dynamics along the river in response to seasonality, physicochemical parameters and geographic distance. While aquatic fungal communities were affected by seasonality and various water pollutants, Ascomycota, Basidiomycota and unclassified taxa were the most dominant groups. Nearly 70% of all identified OTUs represented undescribed species.

We expected higher fungal diversity in less-contaminated areas, and changes in community structure led by the disappearance of the most susceptible OTUs to water pollutants. TRLFP showed that 17% to 18% (dry and rainy seasons respectively) of OTUs present at the source of the river were not found in other sampling locations, indicating that they disappeared or were below the detection threshold of the technique after passing through the city of São Paulo. This result suggests that some fungal groups may be affected by pollutants present in the river, as found by Logares *et al*.^30^ for other microbial taxa. According to Liu *et al*.^31^, fungal diversity diminished when crossing highly-polluted urban areas due the presence of contaminants, confirming that human activities can affect the fungal community. As microbial species may be ecologically classified as copiotrophic (analogous to r-strategist), responding quickly to nutrient availability, or oligotrophic (analogous to k-strategist), showing slower responses and stable population sizes^32^, we hypothesize that the availability of large amounts of organic matter may favor r-strategists and displace the less resilient k-strategists.

Our RDA analysis showed that some physicochemical parameters were important correlates of community structure, although these differed between seasons. Interestingly, this analysis also revealed that the sampled sites were more similar in the rainy season, suggesting a homogenization effect caused by rain. A deeper analysis based on 454 pyrosequencing returned 48,553 OTUs, from which only 11% were shared between seasons. This result confirmed the pattern observed with TRFLP and indicated a clear effect of seasonality in fungal community structure. Sudden environmental disturbances may induce rapid changes in the microbial community structure, making rare groups dominant for a short period of time^33^. The structuring of fungal communities is also likely to respond to the joint influence of environmental and physicochemical variations as well as to biotic interactions induced by nutrient availability^34^. Miura and Urabe^35^ conducted a study to evaluate the effects of dissolved organic matter and nutrients and spatial and seasonal variation in lotic environments on the fungal community. Although they observed that members of the Ascomycota phylum were dominant in environments rich in organic matter, this dominance was dependent on seasonality and temperature, with certain conditions favoring the dominance of the Basidiomycota phylum. Another study assessed the role of the Ascomycota phylum in nutrient cycling in aquatic environments, finding that the presence of these microorganisms was related to the total nutrient content^31^. Taxonomic composition may thus change along the rivers, depending on the environmental conditions and biotic interactions. Our findings corroborate these previous results, as both the Ascomycota and Basidiomycota phyla showed a high relative abundance.

Our diversity and community composition models allowed a finer assessment of the factors underpinning aquatic fungal community dynamics in the Tietê river. The models demonstrated that the physicochemical factors best explaining diversity patterns were pH and dissolved iron, while composition was influenced by BOD, pH, total Al and total Fe, DO, nitrate, TOC, total Fe and seasonality. Highly-contaminated aquatic environments can affect not only community structure but also the metabolic functioning of fungal communities due to the additional stresses caused by pollutants^36^. Previous studies suggested that some of these fungi are adapted to contaminated conditions and can participate in the degradation of toxic compounds at lower pH, facilitating the survival of other species^36,37^. For example, fungi can help mitigate the adverse effects of various compounds by diverse mechanisms, including the production of organic products, chelating, precipitating or binding to heavy metals, retention and immobilization, or absorption of various pollutants such as heavy metals or dyes^38^. Another study described a negative effect of land use on fungal spore diversity, indicating that agricultural practices may cause severe direct or indirect damage to ecological niches available to tropical fungi^34^. In the present study, DO showed a drastic decrease in polluted waters due the abundance of organic matter. Indeed, the discharge of industrial pollutants and domestic sewage in wastewater in the city of São Paulo is likely to have caused a reduction in the concentration of DO. Another study also found a negative correlation between diversity and low DO values, suggesting that the diversity and decomposition processes were affected by limited DO^38^. Finally, BOD showed higher levels in polluted samples, suggesting an effect of organic effluents in specific stretches of the river. On the other hand, lower pH is known to select for acid-tolerant fungi and to reduce overall fungal diversity^39^. Similarly, another study showed that pH affected the number of potentially pathogenic fungi species, indicating that alkalization favored the occurrence of fungi that cause disease in humans and other animals^40^. In our study, sites that presented higher pH, higher total Fe and higher BOD showed enrichment of the OTUs 26177, 9400, 7222, 41193 and 2024, belonging to the families Pleosporaceae (including numerous saprobic, human opportunistic and plant pathogenic taxa) and Trichocomaceae (groups associated with food spoilage and mycotoxin production), as well as other unidentified groups. One study evaluating the capacity of fungi for wastewater treatment reported that remediation capacity was enhanced at lower pH, whereas enzymatic activity increased the production of siderophores, the degradation of toxic compounds and the production of bioproducts of industrial interest. Increasing pH facilitates bacterial growth, causing greater oxygen demand and consequently decreasing the available oxygen in the water. Even though fungi are obligate aerobes, some species can persist in contaminated environments with low oxygen availability, developing opportunistic fungal-bacteria associations^41^.

Seasonality had a strong effect on fungal community composition. This result demonstrates that there was a large turn-over in the aquatic fungal community of the Tietê River, as only a small proportion of taxa (11%) were found in both seasons. We nevertheless caution that we only collected samples in a single year, whereas sampling across several years would be necessary to disentangle the seasonality effect from other temporal fluctuations in water conditions. According to Singh *et al*.^42^ this result suggests that changing environments may be the source of new fungal taxa of biotechnological interest, since environmental variations could expose less frequent groups that are difficult to identify. This rare myco-biosphere may be adapted to transient niches, induced by variations in effluent release in these sites and interactions among various taxa. In addition, we observed that the average water temperature was 22 °C in the dry season and 26 °C in the rainy season. Higher temperatures lead to the reduction of water density, causing a stratification of the water body. As a consequence, the deeper layers remain cold, increasing the availability of nutrients on the warmer surface^43^. Higher precipitation levels compared to the historic average were observed during the rainy season, suggesting that water homogenization could have occurred, reducing the stratification of the water body, consequently reducing the conductivity and BOD levels. Previous studies have also found a similar increase in microbial richness in rivers and reservoirs after precipitation caused by microbial dispersal from the margins into water bodies^44^.

Finally, we detected an increase in community dissimilarity with increasing geographic distance separating sampling locations. The decay of community similarity with geographic distance (known as the ‘distance-decay effect’) has been recognized by ecologists for many decades^45,46^. This decaying effect may be caused by changes in environmental conditions, in turn causing community members to perform different functions (species-sorting), or may be caused by environmental or geographic barriers that could affect dispersal^46^. Whereas aquatic communities of hyphomycetes also showed a decreasing similarity with increasing geographic distance^47^, similar results were also reported for fungal communities of soils in various land use systems^45,48^ as well as in tropical ectomycorrhizal communities^49^. Based on the River Continuum Concept, we argue that the observed pattern may reflect an interplay between hydrological flow conditions, riparian zones, substrate and nutrient availability, dispersal and environmental-led species sorting.

In the present study we explored the factors underpinning the dynamics of aquatic fungal communities in a heavily-contaminated tropical river, highlighting seasonality, physicochemical parameters and geographical distance as the main drivers of diversity and community structure. Our results suggest that water contamination can select for pollution-tolerant and potentially pathogenic groups while reducing the regional species pool. These findings raise potential concerns for human health and the functioning of tropical river ecosystems and they call for improved water sanitation systems in the Tietê River basin. Future efforts are needed to assess the remediation capacity of fungal communities, as has already been done for the bacterial consortium of the Tietê river^50^.

## Electronic supplementary material

Table and figures

Dataset 1
