# Supplementary material for: Influence of water quality on diversity and composition of fungal communities in a tropical river
Source: Sci Rep. 2018 Oct 4;8:14799. doi: 10.1038/s41598-018-33162-y (PMC6172213; doi:10.1038/s41598-018-33162-y)
Supplement: Supplementary file 1 — Table and figures [file 41598_2018_33162_MOESM1_ESM.pdf]

Influence of water quality on diversity and composition of fungal communities in a tropical river

Mabel Patricia Ortiz-Vera, Luiz Ricardo Olchanheski, Eliane Gonçalves da Silva, Felipe Rezende de Lima, Lina Rocío del Pilar Rada Martinez, Maria Inês Zanoli Sato, Rodolfo Jaffé, Ronnie Alves, Simone Ichiwaki, Gabriel Padilla, Welington Luiz Araújo

Supplementary Information

Table S1 Physicochemical characterization of the sampling sites during the dry (13) and the rainy (14) season.

ID	Samplesite	WQI	Conductivity	Turbidity	Temperature	Na	K	NO_3^-	NH_4^+	DO	BOD 5.20	pH	Total Al	TOC.	Dissolved Fe	Total Fe	Total P
1-13	TIET-NASC-13	Great	30	1	18.92	0.0001	4	0.39	0.22	7.51	3	5.8	0.05	1	0.01	0.01	0.02
1-14	TIET-NASC-14	Great	30	1	18.92	0.0001	4	0.39	0.22	7.51	3	5.8	0.05	1	0.01	0.01	0.02
2-13	BQGU03850-13	very bad	1843	71.3	21.78	65	27.5	0.2	31	0.23	120	7.99	10.3	80.9	0.33	3.9	2.4
2-14	BQGU03850-14	very bad	1014	41.5	24.3	75.5	46	0.47	14.2	0.07	48	7.8	5.67	44.7	0.52	2.57	1.2
3-13	TAMT04900-13	very bad	733	47.6	21.33	74.3	13.7	0.2	23.2	0.27	162	7.33	2.22	90.9	2.02	5.4	3.79
3-14	TAMT04900-14	very bad	469	14.5	24.28	47.8	9.5	0.2	9.9	1.1	40	7.21	2.07	28.4	0.32	2.32	1.23
4-14	PINH04100-14	regular	309	101	25.4	13.8	5.6	0.2	4.7	0.7	46	6.8	10.1	17.4	0.4	5.2	0.53
4-13	PINH04500-13	bad	522	25.8	21.61	41.3	10.2	0.2	17.1	1.5	55	6.87	0.55	38	0.44	1.85	2.2
5-13	TIET04200-13	very bad	633	19.2	21.1	56.5	13.2	0.2	19.3	0.58	99	7.24	1.61	49.2	0.43	2.11	2.53
5-14	TIET04200-14	very bad	448	18.7	26.4	42.2	9.88	0.2	9.34	0.71	31	7.14	1.49	27.2	0.97	2.27	0.96
6-13	TIPI04900-13	very bad	590	33.9	19.28	44.4	10.7	0.2	13.1	0.39	96	7.03	3.88	58.8	0.76	3.75	2.3
6-14	TIPI04900-14	very bad	399	21.4	27.7	35.8	8.97	0.2	9.79	0.31	26	7.11	2.05	23.9	1.3	3.12	1.33
7-13	CMDC02900-13	good	196	22	24	14	4.5	2	3	6.5	7	7	1.3	4.76	0.5	2.5	0.3
7-14	CMDC02900-14	regular	142	62	25	19	5	3	0.5	5.4	8	6.9	0.6	9.07	0.3	1	0.3
8-13	TIRG02900-13	bad	646	18.4	21.41	42.3	10	0.2	19.1	0.53	136	7.27	1.45	46.6	0.89	2.94	3.12
8-14	TIRG02900-14	very bad	475	14.7	27.3	32.8	8.63	0.2	12.6	1.02	23	7.28	1.93	20.9	0.5	3.26	1.2
9-13	JUNA04900-13	bad	359	52	22	43	10	3	4	4.7	47	7.2	3	24.7	0.3	2	0.662
9-14	JUNA04900-14	bad	678	47	31	92	18	0.7	7	0.4	96	7.1	0.5	64.6	0.3	0.9	1
10-13	TATU04850-13	bad	737	70.3	21	73	11	0.36	26	0.1	107	7.3	2	10.1	0.7	3	3
10-14	TATU04850-14	very bad	561	49.1	24	56	9	0.08	13	0.3	52	7.2	0.4	41.9	0.3	2	1
11-13	SOIT02900-13	Great	76.6	4.6	22.2	5.5	3	0.2	0.1	8.2	3	8.5	0.1	4.25	0.1	0.16	0.007
11-14	SOIT02900-14	Great	84.5	4.1	19.3	6.45	3.02	0.2	0.21	8.4	2	7.3	0.11	3.89	0.1	0.15	0.02
12-13	SORO02100-13	regular	123.3	12	18.8	16.54	4.05	0.28	0.78	6.2	7	7	0.8	9.51	0.1	1	0.125
12-14	SORO02100-14	regular	156.4	24	26.5	9.92	3.56	0.5	1.1	4.4	6	6.8	1.33	8.85	0.1	1.36	0.17
13-13	CPIV02700-13	bad	209	23	22	26	10	1	3	3.2	34	7.3	17	6.92	0.4	8.5	0.3
13-14	CPIV02700-14	bad	410	24	33	18	5	3	5	2.8	18	8.1	0.3	20.7	0.3	0.3	0.4
14-13	PCAB02195-13	regular	404	8.42	21.3	39	5.5	0.65	4	0.9	5	7.1	1.4	8.98	0.3	1.5	0.6
14-14	PCAB02195-14	bad	706	16.8	18.5	102	11	0.15	6	0.5	20	7.1	0.3	14.4	0.3	1	1
15-13	TIET02400-13	bad	655	17	20.2	45	11	0.1	22.1	0.8	14	7.4	8.53	13.7	0.1	8.12	1.74

15-14	TIET02400-14	bad	264.9	150	25.5	36.9	10.1	2.1	3.15	1.6	36	6.7	12.8	25.4	0.1	7.93	1.27
16-13	TIET02450-13	bad	552	24	20.2	48.15	11.22	0.67	17.9	0.7	13	7.4	7.95	10.9	0.1	7.9	1.36
16-14	TIET02450-14	bad	225	180	26.8	28.2	8.14	1.77	3.2	1.8	33	6.8	8.71	19.5	0.1	4.16	1.26
17-13	JPEP03600-13	good	45	14	20.2	1.35	1.3	0.71	0.1	7.8	2	7.1	0.066	1.48	0.47	1.5	0.025
17-14	JPEP03600-14	good	100	23	25.6	1.29	1.87	1.16	0.28	6.9	2	7.22	0.167	5.7	1.21	2.91	0.08
18-13	TIBB02900-13	good	314	2.74	23.67	31.4	7.01	5.04	0.21	4.98	3	7.1	0.13	5.44	0.1	0.1	0.14
18-14	TIBB02900-14	good	306	8.52	20.29	34.3	7.96	2.92	0.2	9.15	7	8.21	0.1	6.1	0.1	0.1	0.15
19-13	TIET02500-13	good	230	1.76	21.5	29	6.8	3.94	0.1	6.39	3	7.3	0.2	4.14	0.1	0.1	0.06
19-14	TIET02500-14	good	289	4.25	27.95	32.3	7.98	2.31	0.1	2.47	3	6.98	0.13	6.61	0.1	0.18	0.16
20-13	LENS03950-13	good	146	59	21	22	2.38	0.74	0.17	7.4	4	7.4	0.3	4.13	0.48	1.78	0.142
20-14	LENS03950-14	good	196	42	25.1	25.5	2.35	1	0.17	6.8	3	7.4	0.37	7.57	0.738	2.31	0.23
21-13	RGRA02990-13	bad	205	67	22	21.4	3.37	0.47	3.94	5.5	11	7.2	0.4	6.06	0.69	2.31	0.309
21-14	RGRA02990-14	regular	230	31	27.6	15.7	3.29	1	4.88	2.8	11	7.2	0.37	12.3	0.824	2.47	0.79
22-13	JCGU03900-13	good	72	31	24.2	6.78	2.38	1.07	0.1	5.7	2	7.1	0.15	3.89	0.48	1.58	0.087
22-14	JCGU03900-14	good	70.6	25	26.9	4.71	2.85	1.07	0.08	5.5	2	6.9	0.279	7.5	0.863	2.25	0.1
23-13	TIET02600-13	Great	175	2	27.5	24.3	5.02	2.24	0.1	5.7	2	7.3	0.05	4.75	0.06	0.07	0.007
23-14	TIET02600-14	good	237	2.6	28	28.8	7.09	1	0.16	1.2	2	7	0.05	4.35	0.016	0.084	0.02
24-13	ESGT02050-13	good	148	1.4	22.3	15.95	4.5	1.17	0.18	8	2	7.7	0.05	6.84	0.058	0.128	0.007
24-14	ESGT02050-14	good	159	66	28.6	19.3	4.59	1	0.11	8.4	11	8.8	0.05	15.7	0.134	0.184	0.041
25-13	TIET02700-13	Great	149	3.3	22.8	16.65	4.6	1.04	0.1	7.9	2	7.8	0.05	3.35	0.013	0.02	0.01
25-14	TIET02700-14	Great	180	1.6	27.5	20	4.85	1	0.14	5.4	2	7	0.05	3.24	0.013	0.024	0.02
26-13	PATO02900-13	good	95	3.5	21.1	10.04	3.92	1.24	0.1	7.1	2	7	0.121	2.68	0.854	1.7	0.083
26-14	PATO02900-14	good	90	17	25.6	16	4.29	1	0.21	2.4	3	6.6	0.05	10.4	1.5	2.28	0.37
27-13	TITR02100-13	Great	147	1.9	23.9	16.5	4	0.08	0.1	7.5	2	7.7	0.059	3.65	0.016	0.04	0.007
27-14	TITR02100-14	Great	155	2.3	28.5	17.8	4.5	1	0.1	7	2	7.7	0.05	3.25	0.035	0.075	0.02
28-13	TITR02800-13	Great	158	2.2	24.7	18	4.2	0.7	0.1	8.8	2	8.7	0.05	5.19	0.012	0.015	0.007
28-14	TITR02800-14	Great	149.1	1	28	17.9	4.59	1	0.1	5.7	2	7.6	0.05	5.7	0.017	0.013	0.02
29-13	ISOL02995-13	Great	51	1.1	22.7	2.5	1.51	0.33	0.1	7.4	2	7.5	0.05	1.57	0.019	0.02	0.007
29-14	ISOL02995-14	Great	47	1.3	28.3	2.47	1.58	0.34	0.14	7.4	2	7.9	0.05	1.97	0.016	0.026	0.02
30-13	PARN02100-13	Great	78	0.9	24.3	3.41	1.76	0.38	0.12	7.5	2	7.4	0.05	2.67	0.016	0.028	0.007
30-14	PARN02100-14	Great	79.3	1.8	28	3.36	1.74	1	0.1	6.2	2	7.3	0.05	1.49	0.018	0.026	0.02

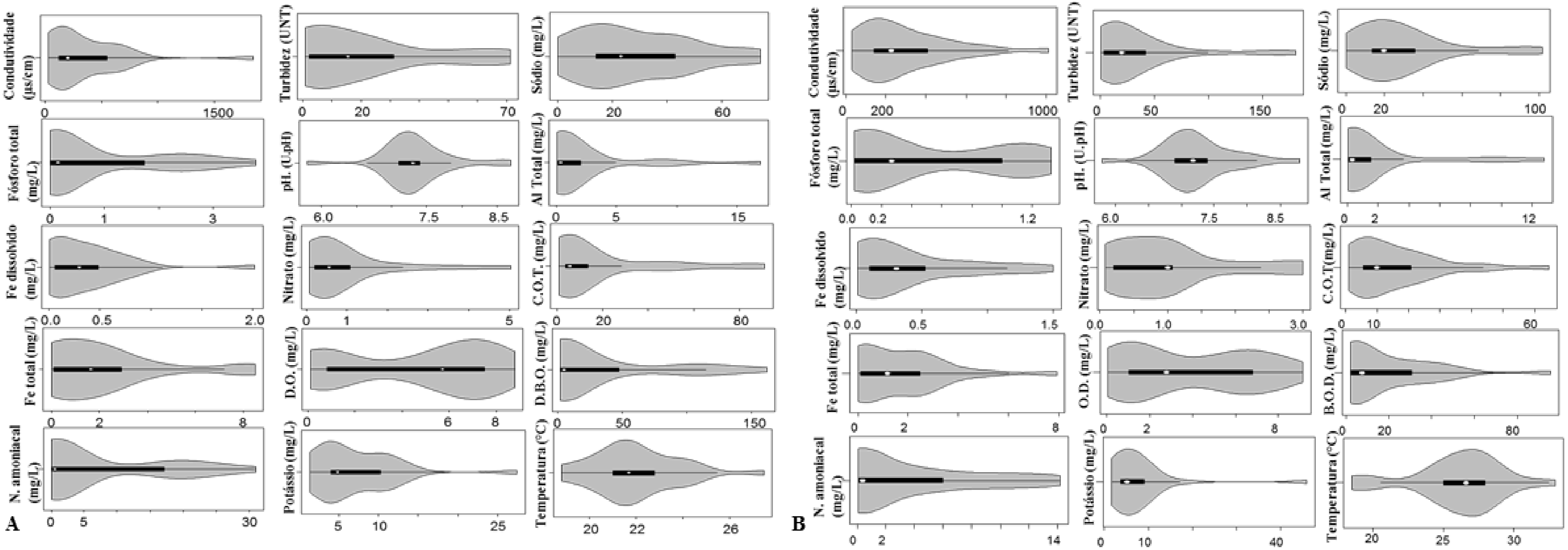

Figure S1 Variation of Physicochemical parameters during the dry (A) and rainy (B) season.

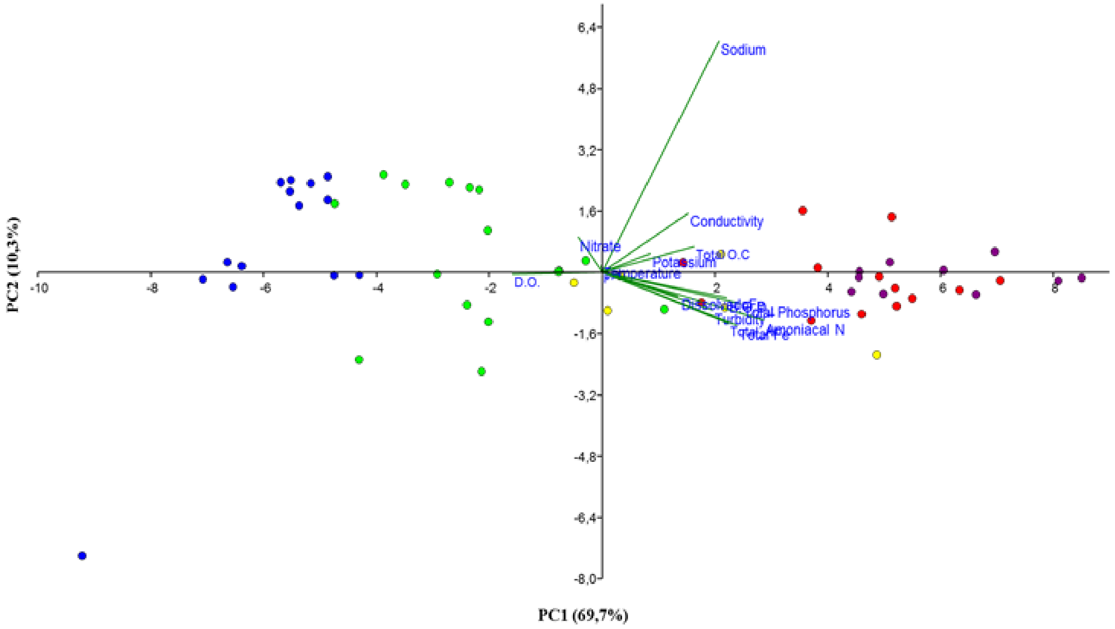

Figure S2 Physicochemical characterization of sampling points by Principal Component Analysis (PCA) for both seasons. Data points are colored according to the Water Quality Index (WQI): blue = Great, green = Good, yellow = Regular, red = Bad, violet = Very bad.

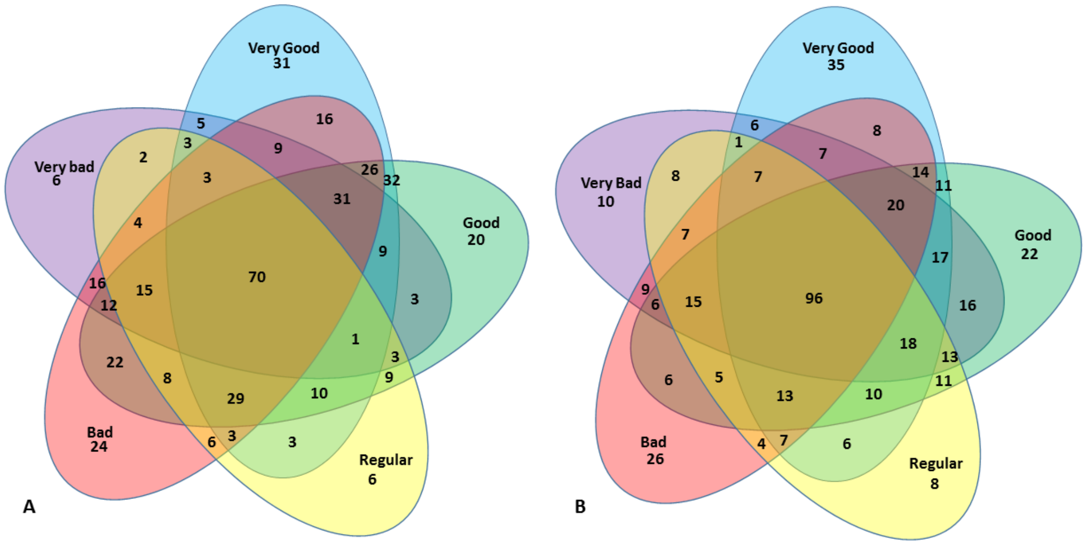

Figure S3: Venn diagram representing the OTUs by Water Quality Index (WQI) during the dry (A) and rainy (B) season.

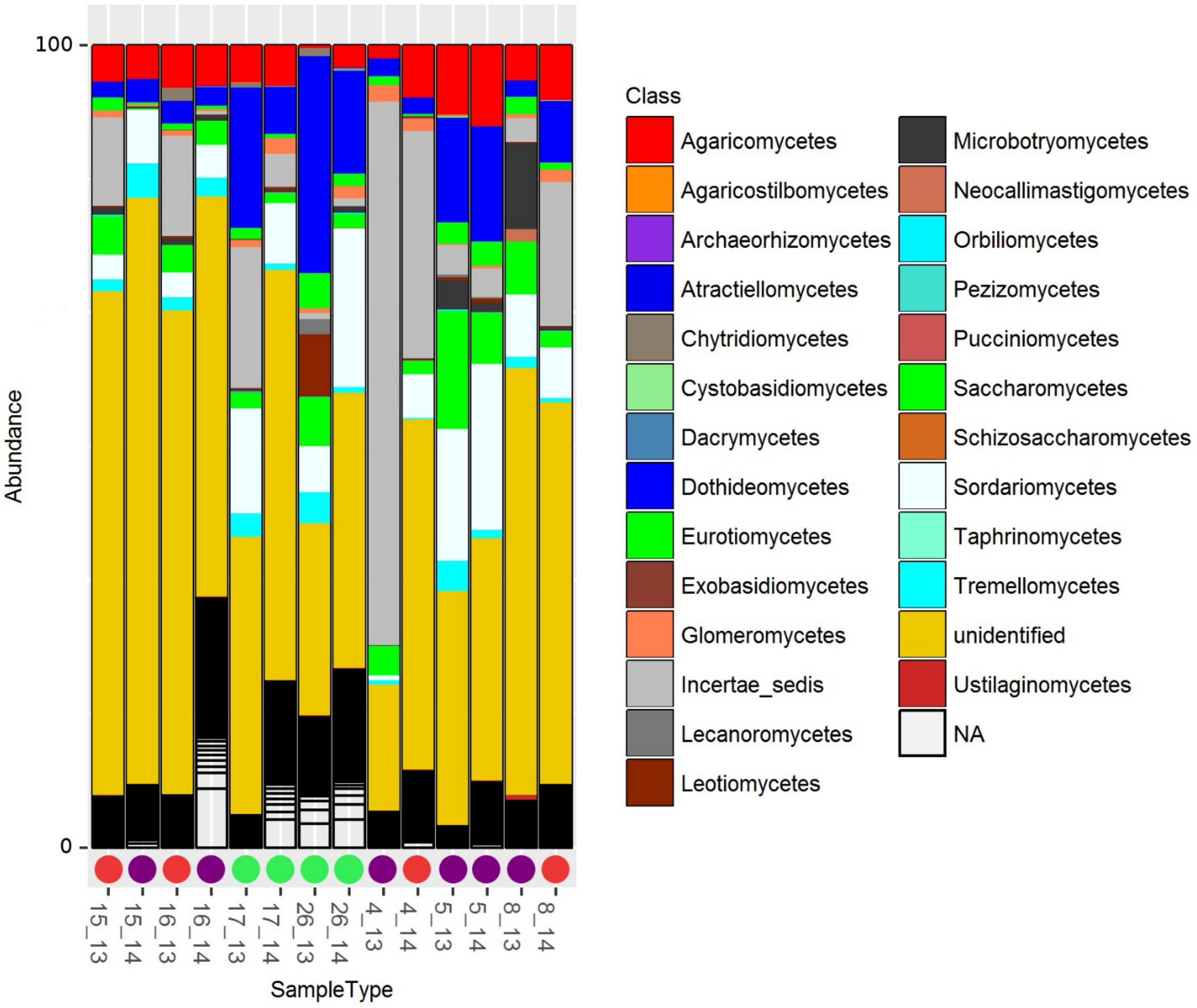

Figure S4: Relative abundances of fungal OTUs. The colored circles at the bottom of the X axis indicate the Water Quality Index (WQI): blue = Great, green = Good, yellow = Regular, red = Bad, violet = Very bad.
